# Parental experience modifies the *Mimulus* methylome

**DOI:** 10.1186/s12864-018-5087-x

**Published:** 2018-10-12

**Authors:** Jack M Colicchio, John K Kelly, Lena C Hileman

**Affiliations:** 1 Department of Plant and Microbial Biology, University of California Berkeley, Berkeley, CA 94710 USA; 2 Department of Ecology and Evolutionary Biology, University of Kansas, Lawrence, KS 66045 USA

**Keywords:** Epigenetics, Methylation, *Mimulus guttatus*, Transgenerational plasticity

## Abstract

**Background:**

Transgenerational plasticity occurs when the environmental experience of an organism modifies the growth and development of its progeny. Leaf damage in *Mimulus guttatus* exhibits transgenerational plasticity mediated through differential expression of hundreds of genes. The epigenetic mechanisms that facilitate this response have yet to be described.

**Results:**

We performed whole genome bisulfite sequencing in the progeny of genetically identical damaged and control plants and developed a pipeline to compare differences in the mean and variance of methylation between treatment groups. We find that parental damage increases the variability of CG and CHG methylation among progeny, but does not alter the overall mean methylation. Instead it has positive effects in some regions and negative in others. We find 3,396 CHH, 203 CG, and 54 CHG Differentially Methylated Regions (DMRs) ranging from tens to thousands of base pairs scattered across the genome. CHG and CHH DMRs tended to overlap with transposable elements. CG DMRs tended to overlap with gene coding regions, many of which were previously found to be differentially expressed.

**Conclusions:**

Genome-wide increases in methylome variation suggest that parental conditions can increase epigenetic diversity in response to stress. Additionally, the potential association between CG DMRs and differentially expressed genes supports the hypothesis that differential methylation is a mechanistic component of transgenerational plasticity in *M. guttatus*.

**Electronic supplementary material:**

The online version of this article (10.1186/s12864-018-5087-x) contains supplementary material, which is available to authorized users.

## Background

Phenotypic plasticity, the ability to modify development according to environmental cues, is of vital importance in response to the constantly changing abiotic and biotic world. While the molecular mechanisms, evolutionary implications, and diversity of plastic phenotypic responses have received considerable attention [1, 2], the ability of parents to transmit signals that evoke plastic responses to the next generation remains poorly understood and skeptically viewed [3]. This skepticism is largely due to ties with “Lamarckism”, but a more scientifically grounded concern is our limited understanding of the mechanisms of epigenetic inheritance. In this study, we utilize whole genome bisulfite sequencing to test how parental damage in the flowering plant, *Mimulus guttatus*, effects the epigenetic profile of the following generation. By utilizing the same *M. guttatus* recombinant inbred line (RIL) in which we previously estimated the transgenerational response of gene expression [4], we are able to identify differentially methylated regions and consider their potential regulation of transposable element (TE) and gene expression.

Conditions experienced by parents have been shown to alter the fitness, phenotype, gene expression, and DNA methylation of progeny for biotic [5–11] and abiotic [12–16] interactions. In *M. guttatus,* progeny plants increase trichome production and differentially express nearly 1000 genes in response to parental damage [4, 7, 8]. The progeny of drought stressed *Polygonum persicaria* alter seedling growth, resulting in increased fitness in dry conditions [17]. Maternal light environment influences offspring growth and increases fitness when offspring environment is similar to parent environment in *Campanulastrum americanum* [14, 18]*.* Evidence from *Solanum lycopersicum* and *Arabidopsis thaliana* suggests that plant hormone response and epigenetic regulatory pathways are vital for at least a portion of transgenerational effects [6]. Yet there remain many open questions regarding the types of loci that are differentially methylated, the magnitude of differential methylation, and the effect of differential methylation on nearby gene expression following from alterations to parental environment [19].

In addition to targeted loci or phenotypes that are modified by parental environment, there also appears to be a general increase in the variability of the epigenome in response to stressful parental conditions. Response to a wide variety of parental environmental signals in the progeny of apomictic dandelions (*Taraxacum officinale*) increases DNA methylation variation [16] and alters a wide array of progeny phenotypes including root:shoot ratios and specific leaf area [20]. While effects of parent environment on the mean DNA methylation of a locus has been the primary class of transgenerational effect studied thus far, it is also possible that modifying the stochasticity of the epigenome is beneficial in harsh or changing environments.

DNA cytosine methylation, histone modifications, and small RNAs are key epigenetic regulators, that act through linked pathways [21–24] to alter the transcription and translation of genes. DNA methylation in plants can occur on any cytosine nucleotide, but the mechanisms that propagate methylation and the effects of methylation on genome regulation vary between those in CG, CHG, or CHH methylation contexts, where H is any nucleotide other than G [25–28]. While all three types of methylation act in silencing TEs, their role in regulating gene expression is more variable. Both the methylation context and the location of methylation relative to coding sequences (upstream, downstream, within introns/exons) affect the relationship between methylation and gene expression [29, 30].

CG methylation is found at the highest frequencies in flowering plant DNA—occurring on over 50% of CG sequences in many species [31]. CG methylation upstream of gene coding sequences is associated with suppression of gene expression [28, 29, 32]. CG methylation in gene coding sequences tends to be relatively modest in flowering plants, except in *M. guttatus* where it is present at moderate to high levels [33], and its effects on gene expression are complicated and disputed [29, 31, 34, 35]. From work in *M. guttatus* and other systems it appears that interactions between gene length, upstream DNA methylation, and other factors alter the role, if any, of coding sequence CG methylation on gene regulation [28, 29, 36].

CHG and CHH methylation are often grouped as “non-CG” methylation, and occur at significantly lower levels across plant genomes [29, 33, 37]. CHG and CHH methylation are propagated and reiterated by partially overlapping pathways, often initiated by 24-nt small interfering (si) RNAs [23, 24, 38]. Non-CG methylation is associated with transcriptional repression. While epigenetic marks such as histone modifications appear to be reset during gamete formation in plants [39] and many methylation marks are reset during embryo development [40], the recent discovery that si-RNAs are loaded into pollen granules [41], are phloem mobile [38], and can mediate methylation in recipient cells [42] presents one possible mechanism through which environmentally induced epigenetic marks may be transmitted between generations [43]. Additionally, a growing body of work utilizing epiRILs and other approaches have demonstrated that methylation patterns are highly heritable (see review [44]), providing evidence that altered epigenomic profiles can be inherited across generations.

Holeski (2007) [8] demonstrated that parental leaf damage can induce a transgenerational response of elevated trichome production in *M. guttatus*, and that the response is variable among genotypes. *M. guttatus* is therefore and excellent model for studying the ecology and evolution of transgenerational plasticity. Since Holeski (2007), we have determined that induction can be transmitted both paternally and maternally, is partially dependent on DNA methylation, and persists through at least two generations [45]. The transgenerational signal initiated by parental leaf damage in a highly responsive genotype induces the differential expression of nearly 1000 genes in progeny [4]. Transgenerationally plastic responses resulting from parental leaf damage in *M. guttatus* have significant effects on plant resistance to herbivory in the field [46], and may involve additional plant defensive responses beyond trichome induction (*e.g.,* general stress response; [4]).

Here, we investigate how parental environment alters the offspring methylome. We carry out our experiments in recombinant inbred line (RIL) 94, a model genotype for transgenerational plasticity. RIL-94 exhibits a strong induction phenotype in which offspring of leaf damaged plants mount a defensive response including increased trichome production [8]. Working in this isogenic background minimizes phenotypic, transcriptomic, and epigenetic variation, allowing us to better characterize the transgenerational response, compare results across independent experiments, and in the long-term explore transcriptome and epigenome responses across inductive and non-inductive *M. guttatus* genotypes. We present whole-genome methylation data from progeny of damaged and control *M. guttatus* RIL-94 plants, and examine patterns of differential methylation in relation to previously published gene expression results from our RIL-94 model.

**Fig. 1:**
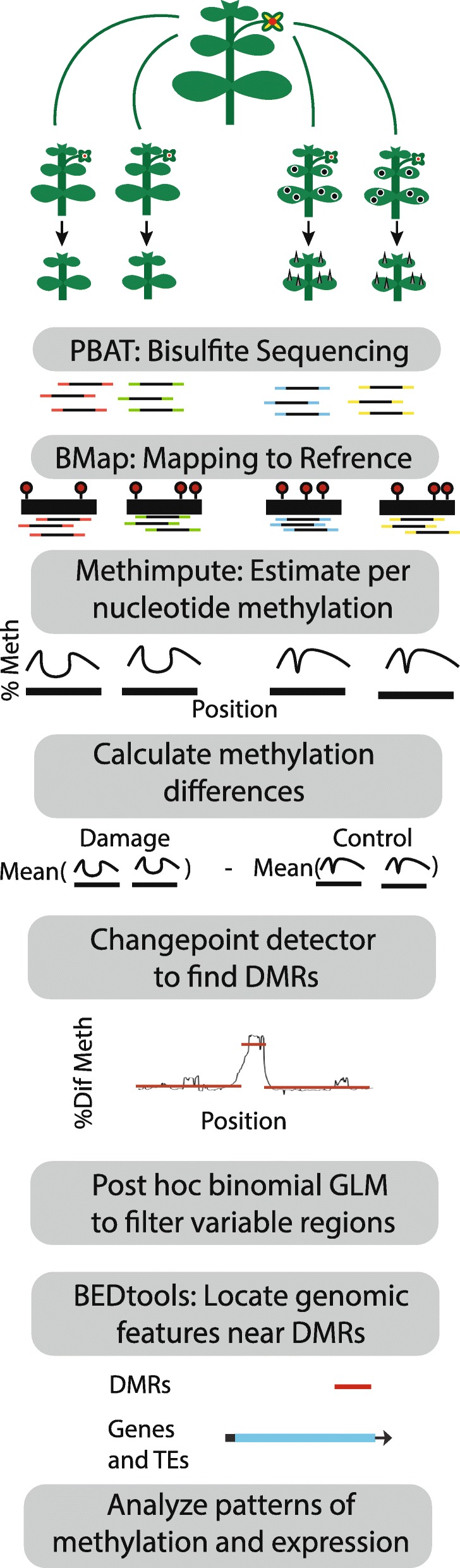
Schematic of the pipeline used to study differential methylation resulting from parental environment in *Mimulus guttatus*

We implement a novel computational methodology to more accurately estimate individual cytosine methylation levels based on the methylation of nearby sites, followed by a unique application of the PELT changepoint detector algorithm [47], and a generalized linear model framework to identify regions of consistent differential methylation (Fig. 1). Within this framework we address whether parental environment influences global or locally targeted DNA methylation patterns. We test whether different classes of methylation (CG, CHG, and CHH) vary in their response to parental environment, and which genomic features appear to be regulated by these different classes of methylation. Lastly, we test the hypopthesis that stressful environmental conditions lead to increased epigenetic variation.

## Methods

### Experimental Treatments and Tissue Collection

Selfed seed from a single *Mimulus guttatus* RIL-94 plant (F_8_ generation) was split randomly and grown into damage and control treatment groups as in Colicchio *et al.* [29]. Briefly, F1s was generated as a cross between an inbred line, IM 767 derived from a high elevation annual population at Iron Mountain, Oregon, and a single plant from the coastal perennial population at Point Reyes National Seashore, CA. Details on the collection, identification, and selfing of IM 767 can be found in Willis, 1999 [48], and detailed information on the generation of the RILs can be found in Holeski, 2007 [8]. Briefly, A single F1 from this cross was self-polinated to generate 1000 F2 individuals. These lines were than propagated through single seed descent for eight generations to create nearly isogenic F8 lines [8]. RIL-94 was found to be a consistent inducer of trichome production in response to parental wounding, and therefore has been used as a model genotype in our previous analysis of transgenerational gene expression plasticity [4], and now here. For damage treatment, we punched two holes of ca. 6mm diameter in each leaf at the developmental point when the next leaf had expanded. While mechanical wounding does not elicit the full suite of plant herbivory responses, prior transgenerational phenotypic and gene expression work has demonstrated that *Mimulus* responds in a relatively strong and consistent fashion to mechanical wounding making it a tractable and efficient means of inducing plant responses [4, 8]. Five damaged and five control plants were self-pollinated to propagate seed to determine transgenerational effects. Progeny of damage and control individuals were grown until the second leaf pair expanded to one centimeter in width, at which point each leaf from the second node was flash frozen in liquid nitrogen.

### DNA extraction, library preparation, and read mapping

We extracted DNA from leaf tissue using a CTAB protocol [49] and generated libraries for whole genome bisulfite sequencing (WGBS) following the PBAT (Post-Bisulfite Adaptor Tagging) protocol [50]. With 1 ng of unmethylated lambda DNA (Promega) used as a spike-in control for conversion efficiency, 55 to 100 ng of genomic DNA from each individual was bisulfite treated (EZ DNA Methylation kit, Zymo Research). We performed two rounds of random primer extension for tagging bisulfite treated DNA with adaptors using primers for paired-end library construction. Unique adaptors were ligated onto each library to allow for down-stream de-multiplexing. Template concentrations were determined by qPCR using Library Quantification Kits (KAPA biosystems). Due to low library quantities, one control individual was omitted from sequencing. We sequenced these libraries (in combination with three other PBAT WGBS libraries; 12 samples per lane) on two HiSeq 2500 rapid-run 150bp paired-end lanes at the University of Kansas Genome Sequencing Core. Each library was sequenced on both lanes.

We constructed a RIL-94 reference genome, re-annotated genes (Additional file 1: Table S1) and transposable elements (Additional file 2 Table S2), mapped our WGBS libraries to this reference using bmap [51], and used methimpute to impute methylation across the genome more accurately [52] (Additional file 3: Methods S1).

### Variance Comparison

To compare methylome variability between treatment groups, we calculated the within treatment group variance for methylation at each cytosine. Additionally, we calculated the mean damaged and control group variability within each gene coding region. We separated methylation based on context (CG, CHG, CHH) used a paired t-test to compare patterns of within group variation. We used ANOVA to consider the effect of within group methylation variation on previously identified (Colicchio *et al.* [29]) within group expression variation.

### Identification of differentially methylated regions

We subtracted mean parental damage from mean control methylation at each cytosine to estimate site-specific difference in methylation between treatments. We separated site-specific differences in mean methylation by sequence context and converted them into three independent vectors for each context, which we then split into 14 vectors, one for each chromosome. Each vector was entered into the R package “changepoint” (2.2.2) using the pruned exact linear time (“PELT”) [47] algorithm and a manual penalty of 1.4. This more stringent penalty decreased false positives compared to the penalty of 1 used in the initial methylome PELT paper [51]. The PELT changepoint detection algorithm was designed to identify changepoints (here, in methylation difference between the two treatment groups) in large datasets where the computational demands increase only linearly with the number of observations. Using PELT changepoint detection across the *Mimulus* genome, we identified base positions where there is a shift from near-zero to larger differences in methylation between treatment groups due to parental environment. We only considered regions with at least a 4% change in methylation between treatment groups going forward.

To confirm that PELT-based assessment of differential methylation was both consistent across samples within a treatment, and not wholly driven by imputed data rather than observed methylation calls, we summed the observed number of mapped unmethylated and methylated cytosines for a given context within each PELT-defined region for each individual. Next, we used a generalized linear model (glm) approach to find regions that had a significant effect of parental treatment on offspring methylation. We performed this analysis using the R package “lme4” (1.1) function glmer with a binomial family and “logit” link function to test for differential methylation within candidate regions [53]. We treated each mapped cytosine’s methylation state as a binomial response variable, with parental treatment as a fixed effect, and individual as a random effect. While we used quantitative data for % methylation in the methimpute/PELT approach to construct the methylome and contrast mean methylation between the damaged and control group, within the glm framework, we returned to the raw data to confirm consistent differential methylation between groups. This allowed us to explicitly test whether within a putative DMR region there is a significant effect of parental treatment on the frequency of methylated relative to unmethylated cytosines. FDR adjusted p-values were calculated with the R package p-adjust using the Benjamini-Hochberg correction (method=“BH”); FDR adjusted p-values less than 0.05 were considered differentially methylated regions and retained for downstream analyses. As this FDR correction is preformed on regions originally identified on the basis of their large mean differences, it must be noted that due to the filtering of regions prior to testing, approximately half of the genomic segment were not considered in the glmer, and in turn not included in our FDR correction. For this reason the actual FDR could more conservatively be considered 0.1, accounting for the fact that the total number of statistical tests preformed without this previous filtering step would have been approximately two times greater, leading to approximately two times as many potential false positives in our set of candidate genes. Still this FDR correction is useful in limiting our following analyses to a more confident set of DMRs.

The PELT changepoint detection approach was previously successful in fragmenting an individual methylome into “methyl-regions” [51]. Here we extended this approach to identify DMRs between groups of individuals. While the mathematical methods and tools utilized vary greatly between our approach and the recently developed R package “dmrseq” [54], the two approaches share the concepts of first identifying candidate DMRs (here through methimpute and “PELT” changepoint detection), followed by explicit hypothesis testing within these regions (here using a binomial glm framework). Our pipeline is publically available on Github (https://github.com/Methylflower/DMR-scan) and includes both the code necessary to run this pipeline on real methylome data as well as a simulation framework to test this pipeline. Using this simulation framework we generated methylome data with areas of known differential methylation, and others with stochastic noise but no significant differences in methylation probability between treatment groups. Our pipeline successfully identified significantly differentially methylated regions, and was able to filter out regions with high variance but no consistent differences in methylation.

### Annotation of DMRs

We used BEDtools [55] “nearest” to identify overlapping or nearest genes and TEs to each DMR. To determine whether different DMR contexts overlap with different genomic features, we constructed contingency tables with groupings based on the DMR context and the presence/absence of a coding region, regulatory region (+/- 2kb from coding regions), or TE. To determine the classes of genes that tended to be differentially methylated, we preformed GO enrichment analysis of genes overlapping with CG DMRs and non-CG DMRs using the PlantRegMap server [56]. To preliminary assess the role of differential methylation on gene expression, we constructed contingency tables with groupings based on the presence/absence of an overlapping DMR with whether or not the gene was previously identified as differentially expressed (from Colicchio et al., 2015b). Chi-square tests were performed for these contingency tables. Additionally, we performed these same tests for genes that did not overlap DMRs, but were within 2kb of a DMR.

## Results

Using WGBS we sequenced the methylomes of five individuals derived from parents exposed to mechanical damage, and four individuals of control parents (SRA: SUB4505793 ). We mapped bisulfited reads to the newly constructed RIL-94 reference genome, and obtained a mean read depth of between 6 and 9.5 (Table 1). Conversion rate was between 98.5 and 99.4 percent (calculated on lambda control DNA) and was not found to be associated with mean methylation percents across the genomes of our samples. Mean methylation was slightly higher in the offspring of damaged than control plants (Table 1). Differences in mean methylation were largely due to the offspring of one control plant (OC2) with low methylation in all sequence contexts. We constructed methylation domain landscape plots (Additional file 3: Figure S1) for CG methylation based on change-point detection analyses that break down the genome into regions of relatively consistent methylation. Plot results suggest that global methylation patterns are not altered in the offspring of damaged compared to control plants. In both damaged and control progeny two types of methylated regions predominate; large 1kb-20kb regions of 75%-95% methylation, and smaller 250bp-5kb regions of less than 20% methylation. Confirming our previous findings in *M. guttatus* (Colicchio *et al.*, [4]) and from other systems [28, 31], we found that CG methylation peaks near the 3’ end of genes, the lowest CG methylation is often directly proximal transcriptional start sites, and CHG and CHH methylation tend to be elevated directly upstream and downstream of coding regions (Additional file 3: Figure S2).

**Table 1:** Genome-wide percent methylation in offspring of leaf-damaged (OD) and offspring of control (OC) *M. guttatus*

Individual	Mapped Basepairs	Average Read Depth	%CG	%CHG	%CHH
OC1	1,888,902,727	6.4	70.4	35.9	14.9
OC2	1,877,864,952	6.4	58.9	26.8	11.6
OC3	1,867,678,993	6.3	67.6	30.6	12.3
OC4	1,778,153,365	6	70.0	36.8	13.9
OD1	1,219,264,220	4.2	73.1	40.3	14.2
OD2	1,860,074,372	6.3	72.8	39.9	14.3
OD3	2,386,764,313	8.1	72.9	39.5	14.1
OD4	1,941,206,754	6.6	71.2	39.4	16.3
OD5	2,797,016,391	9.5	69.0	34.2	14.0

### Methylation Variability

**Fig. 2:**
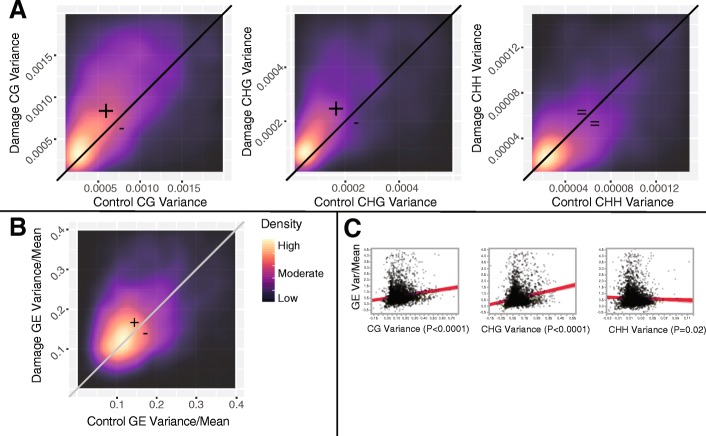
Shifts in the variability of methylation and gene expression in response to parental damage, and the relationship between methylation and gene expression variability. **a** Density plot of within damage treatment group variance (Y-axis) relative to within control treatment group variance (X-axis) for gene body CG, CHG, and CHH methylation. **b** Density plot of within damage treatment group variance (Y-axis) relative to within control treatment group variance (X-axis) for gene expression [4]. **c** Relationship between the within treatment group methylation and gene expression variance of a given gene

Comparing the within treatment, between individual variation of methylation at individual cytosines, we found the progeny of damaged plants were more variable genome wide for both CG and CHG methylation (paired t-tests, *p* <0.001; Table 2, Fig. 2a). This pattern was most evident for nucleotides within gene coding regions (Table 2). On the other hand, there was a slight decrease in inter-individual CHH variability in response to parental damage.

**Table 2:** Levels of within treatment methylation variation, both genome-wide and restricted to coding-regions

	Genome-Wide		Coding Region	
	Control	Damaged	Control	Damaged
CG	0.0062	0.0066	0.0050	0.0063
CHG	0.0064	0.0070	0.0030	0.0035
CHH	0.00020	0.00018	0.00013	0.00012

Previously collected gene expression data demonstrated that *M. guttatus* RIL-94 offspring of damaged parents also have significantly higher expression variability compared to control offspring (Colicchio *et al.* [29]) (Fig. 2b). We found a significant relationship between the within treatment group methylation variability of a gene and its gene expression variability. Genes with elevated CG and CHG methylation variation in this study exhibited elevated gene expression variation in Colicchio *et al.* [29] (CG: t=10.18, effect: 1.24, *p* <0.0001; CHG: t=2.49, effect: 2.50, *p* <0.0001; Fig. 2c), while CHH methylation variation was inversely associated with previously identified gene expression variation (CHH: t=-2.45, effect:-2.12, *p*=0.014; Fig. 2c). This pattern suggests that higher inter-individual CG and CHG methylation variation may be associated with elevated gene expression variation in response to parent leaf damage.

### Specific genomic regions experience CG, CHG, and CHH differential methylation

**Fig. 3:**
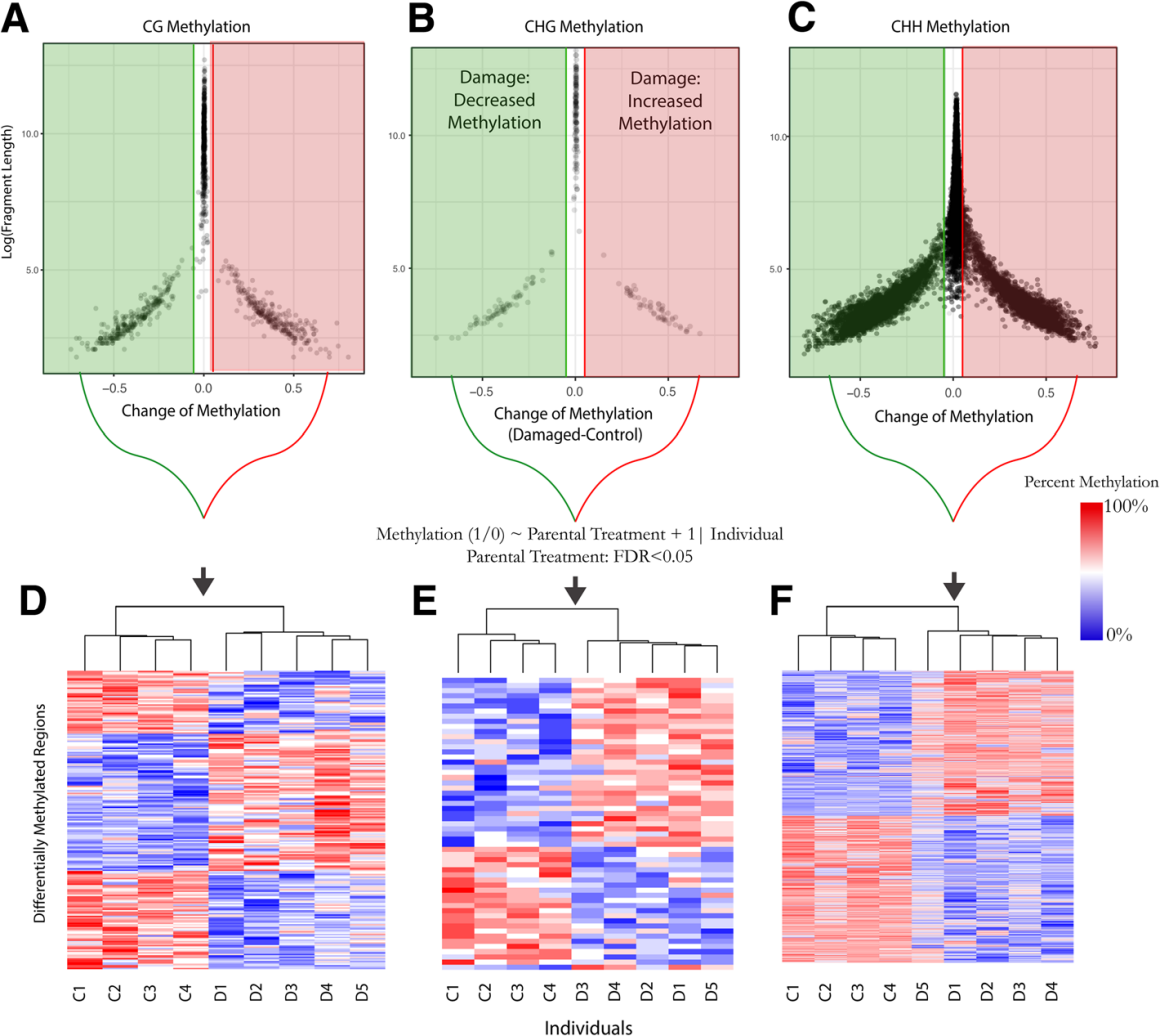
Differentially methylated genomic regions at CG, CHG, and CHH contexts based on PELT and GLM analyses. In the top panel (**a-c**) PELT-derived methylation difference (parental damage - control) are reported as a function of imputed size (log-transformed). For each context, there is a general pattern of larger regions with little change to methylation in response to parent damage (white), and smaller regions with either higher methylation levels in control samples (green) or higher methylation levels in progeny of damaged parents (red). **d-f** Regions of significant differential methylation across the three contexts (up or down, FDR<0.05 based on GLM approach) are shown in heatmaps with percent methylation standardized by regions

To determine whether there were any regions of the genome with distinct methylation patterns dependent on parental environment, we utilized a combinations of tools to first smooth across the methylome (methImpute: [52]), then scan for regions with similar or different levels of methylation between treatments (PELT changepoint detection: [57]), and finally test whether this response is consistent across replicates (lme4 binomial glm: [53]). For CG, CHG, and CHH sequence contexts, we found that the genome was composed primarily of large regions with relatively low differences in mean methylation between the offspring of damaged and control individuals (Fig. 3a-c). However, in all contexts, the PELT changepoint detector algorithm identified smaller regions with substantial differences in mean methylation between the two treatment groups (Fig. 3a-c).

**Fig. 4:**
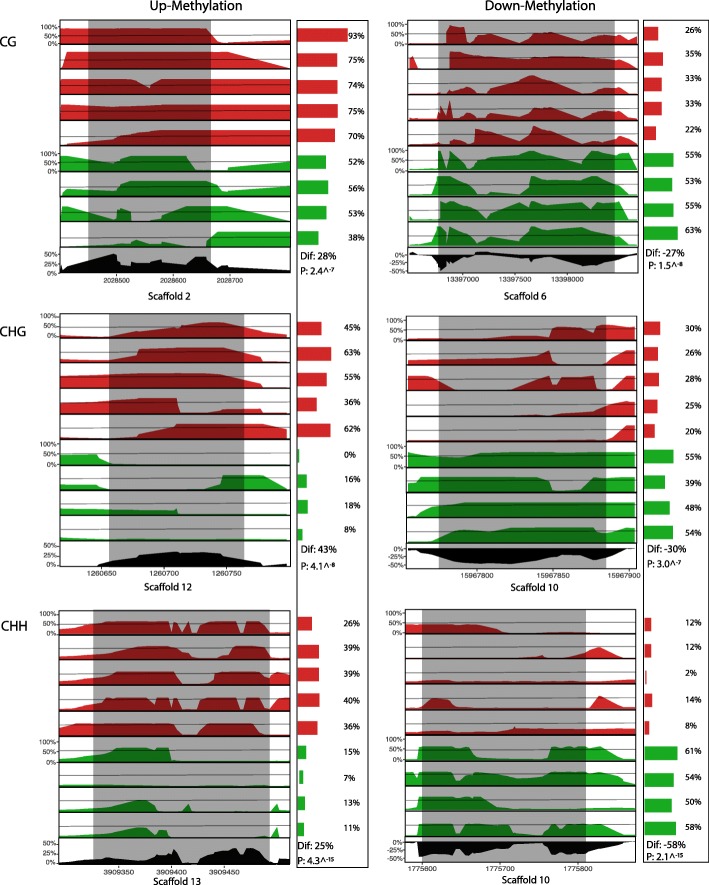
Visualization of PELT methylation patterns across representative regions identified as differentially methylated (shaded regions) for the nine individuals sequenced in this study: five offspring of damaged individuals (top; red), and four control offspring (bottom; green). Bars and percentages to the right of methylome plots show the percent of methylated cytosines mapped in that region for each individual. The Black track at the bottom of each panel shows the mean difference in percent methylation across this portion of the genome. “Dif.” = Percent mean difference between treatments, and “P” = P-Value of generalized linear model comparing distribution of methylated and unmethylated cytosines between treatments

Of the candidate DMRs that met our criterion of 4% difference in methylation, our glm approach parsed out DMRs exhibiting consistent patterns of differential methylation in response to parental treatment (Fig. 3d-f; FDR < 0.05) from those DMRs with variable methylation not associated with parental treatment. After filtering genes based on this criterion, we found 54 (of 127) CHG, 203 (of 452) CG, and 3,396 (of 8,356) CHH DMRs (Fig. 3d-f, Additional file 4: Table S3). CHG DMRs were on average the largest (88 to 6,991bp, mean: 851bp, median: 543bp), followed by CG DMRs (17 to 9,879bp, mean: 504bp, median: 222bp), and CHH DMRs (22 to 15,457bp, mean: 274bp, median 146bp). Clustering of individuals based on patterns of methylation within DMRs confirmed that across all contexts, offspring of damage individuals were more similar to each other than to offspring control individuals (Fig. 3d-f). Six DMRs representing the full factorial combination of methylation class and direction change of methylation were chosen to provide a visualization of the spatial and inter-individual variation in methylation across DMRs (Fig. 4).

The largest mean changes in methylation was in CG DMRs (6.5% to 74.5% change, mean: 38.7%, median: 38.6%), followed by CHH DMRs (4.0% to 67.0% change, mean: 31.0%, median: 31.3%), and CHG DMRs (12.3% to 52.2% change, mean: 30.7%, median 30.2%) (Additional file 3: Figure S3). We found slightly more up-methylated than down-methylated CG DMRs in response to parental damage (110 up, 93 down), with the opposite for CHG DMRs (22 up, 32 down), and nearly equal direction of change for CHH DMRs (1693 up, 1703 down). For all DMR contexts, larger DMRs tended to have a smaller absolute difference in methylation (*p* <0.0001; Fig. 3a-c). CG and CHG DMRs showed no significant effect of the size of the DMR on the direction change in methylation, while CHH up-methylated DMRs were significantly larger (mean: 344bp) than CHH down-methylated DMRs (mean: 201bp, *p* <0.0001, Fig. 3c).

### Damage induced changes in non-CG methylation is associated with transposable elements

**Fig. 5:**
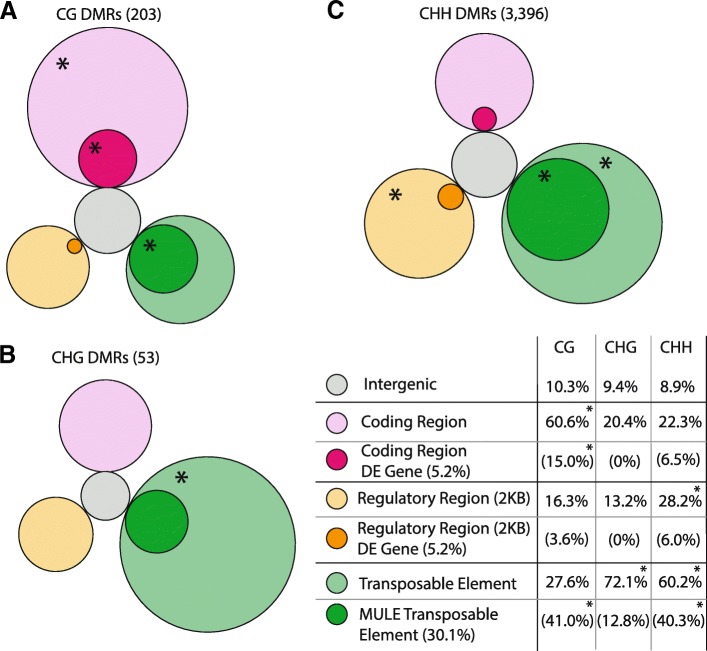
Circular diagrams showing the relative proportion of CG, CHG, and CHH DMRs across different genomic contexts. Intergenic regions are classified as those that do not fall into the other three categories, while three other categories (coding region, regulatory region, and transposable element) are depicted as the outer circles. Inner-circles represent subset of the DMRs within that group: (1) Magenta: DMRs that overlap coding regions that were found to be differentially expressed in Colicchio, 2015b (2) Orange: DMRs that are within 2kb up-stream or down-stream of genes that were previously found to be differentially expressed in Colicchio, 2015b (3) Dark green: DMRs that overlap the MULE class of transposable elements. Asterisks in outer circles depict genomic contexts for which DMRs of that sequence context are enriched relative to other sequence contexts. Asterisks in inner circles represent classes enriched relative to the genome-wide average. **a** CG DMRs, **b** CHG DMRs, **c** CHH DMRs

**Fig. 6:**
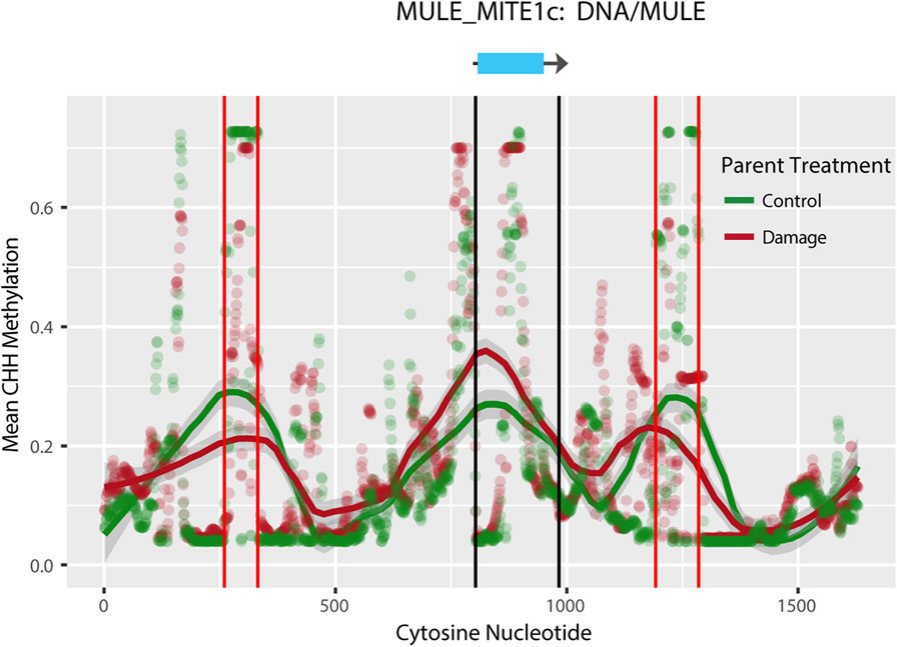
CHH Methylation patterns in and around a MULE transposable element. This pattern is one of the most commonly observed for MULE elements in this study, with elevated CHH methylation overlapping the transposable element, and regions of decreased methylation flanking the TE

Non-CG DMRs overlapped most frequently with TEs (Fig. 5, Additional file 3: Figure S4). Only 56 (27.6%) CG DMRs overlapped with TEs, while 2103 (60.4%) non-CG DMRs overlapped with TEs (X^2^=91, *p* <0.0001, see Fig. 6 for an example of CHH DMR overlapping TE). There was a slightly, but not significantly higher frequency of CHG DMR TE overlap (72%) than CHH DMR TE overlap (60.2%, X^2^=3.2, *p*=0.07). Only considering CHH DMRs due to the higher sample size, we found that DMRs up-methylated in response to parental damage overlapped with TEs (69%) more frequently than down-methylated DMRs (51%, X^2^=110.1, *p* <0.0001). We found a significant effect of TE class on overlap with CHH DMRs, even after accounting for TE size (X^2^= 298, *p* <0.0001). Mutator-like element (MULE) TEs (Class II TE) were most highly enriched in the set of DMR overlapping TEs (MULE: 41% of DMR TEs, 30% genome-wide, Figs. 5 and 6).

### Differentially CG methylated regions overlap with gene coding regions

Of the 203 CG DMRs, 123 (61%) overlapped with gene coding regions, as did 11 (20%) CHG DMRs and 763 (22%) CHH DMRs (Fig. 5, Additional file 3: Figures S4 and S5). A Chi-Square contingency table test confirmed a significant effect of DMR context on probability of overlap between a DMR and a coding sequence (X^2^=150.77, *p* <0.00001). An additional 33 (16%) CG DMRs, 7 (13%) CHG DMRs, and 957 (28%) CHH DMRs were within 2kb upstream or downstream of a coding sequence (Additional file 4: Table S3, Fig. 5). The effect of DMR context on presence of DMR within 2kb of a gene coding region (X^2^ =18.845, *p*=0.000081) suggested that different DMR contexts may also be present at different frequencies around gene regulatory regions, with CHH DMRs most often located within 2kb upstream or downstream of gene coding sequences (Fig. 5). CHH DMRs with lower methylation in response to parental damage were significantly closer to genes (median: 1.5kb) than CHH regions with higher methylation (median 2.4kb) in response to parental damage (*p* <0.0001). Direction of methylation change did not vary with respect to distance between gene and DMR for the other two methylation contexts.

Among other terms, gene ontology enrichment analysis suggests that genes involved in regulation of hormone levels (*p*=0.00019), oxidoreductase activity (*p*=0.0005), and response to external stimulus (*p*=0.01103) were enriched in the set of genes overlapping CG DMRs (Additional file 5: Table S4). Non-CG DMRs were enriched for GO terms auxin polar transport (*p*=0.00039), palmitoyl hydrolase activity (*p*=0.000016), protein binding (*p*=0.0015) and hormone transport (p=0.00131) (Additional file 5: Table S4).

### CG DMRs may overlap with previously identified differentially expressed genes

While our current data are not sufficient to evaluate the effect of differential methylation on differential expression because expression data for the same experimental design were collected on a different set of RIL-94 plants, a preliminary contrast is still useful (see *Discussion* for rationale). We tested whether genes associated with DMRs were enriched for the set of genes previously found to be differentially expressed in *M. guttatus* RIL-94 leaf tissue of damage compared to control offspring (Colicchio *et al.* [29]). Because we only considered genes from an older annotation used for the gene expression study (Colicchio *et al.* [29]), this analysis was limited to 804 genes: 107 (of 123) CG DMRs, 9 (of 11) CHG DMRs, and 688 (of 763) CHH DMRs overlapping gene coding sequences in the previous annotation (Additional file 3: Figure S6). Sixteen of the 107 (15.0%) genes with overlapping CG, 0 of the 9 genes with overlapping CHG, and 45 (6.5%) of the 688 genes with overlapping CHH DMRs were differentially expressed by at least two methods in Colicchio *et al.* [29] (Fig. 5, Additional file 3: Figure S5). Of the remaining genes that do not have coding region-DMR overlap, 5.2% were previously found to be differentially expressed by at least two methods. A Chi-Square Contingency table test found the distribution of differential expression to be uneven between gene coding sequences associated with CG DMRs, non-CG DMRs, and genes coding sequences not associated with DMRs (X^2^=21.55, *p*=0.000021). Enrichment of differentially expressed genes overlapping with CG DMRs explained the majority of the Chi-Square value (19.22/21.55); nearly 3 times as many genes overlapping with CG DMRs were found to be differentially expressed (16) as expected by chance (5.6). Two of these sixteen genes that were found to be both differentially methylated and expressed are visualized in Additional file 3: Figure S7. We found no evidence for enrichment of differentially expressed genes in the set that overlapped with regulatory regions for CG (1/28: 3.6%) , CHG (0/4), or CHH DMRs (51/844: 6.0%, *p* >0.1).

## Discussion

Parental damage in *M. guttatus* produces a signal that is transmitted to progeny and ultimately alters gene expression and phenotype [4, 7, 8, 45]. Here we expand our understanding of transgenerational plasticity in *M. guttatus*, identifying both genome-wide increases in epigenetic variability and localized regions of differential methylation between progeny of damaged and control parents. Below, we discuss the observed increases in epigenetic variability, how the transgenerational response in methylation differs by cytosine context in both nature and consequence, and the potential adaptive significance of transgenerational methylation as a response to stressful conditions.

### Parental damage increases offspring methylation variance

DNA methylation variation increased in the offspring of damaged relative to control plants (Fig. 2). This genome-wide pattern was most pronounced for CG methylation (>25% increased variation across coding sequences). Previous gene expression work identified a coinciding increase in gene expression variation in progeny of damaged plants (Colicchio et al. [29]). This parallels results from asexual dandelions where the offspring of plants exposed to salt stress or various plant hormones exhibit increased epigenetic diversity (Verhoeven et al., [16]). Taken together, these results provide substantial evidence that the offspring of plants exposed to stressful environments may exhibit more epigenetic, gene expression, and potentially phenotypic variation than the offspring of plants grown in the absence of environmental stresses.

The transmission of increasingly variable epigenomes in the face of environmental stress may widen the distribution of progeny phenotypes in a potentially adaptive manner. Changing environmental stresses can increase the distance between an organism’s phenotype and the optimal phenotype for their progeny. Some organisms may plastically respond by altering phenotype in a specific direction. However, in situations where an organism is facing novel stresses, increases in offspring phenotypic variability may also increase fitness [58]. Increased epigenetic variability across offspring may generate a wider range of progeny phenotypic diversity, possibly increasing fitness in an environment that was stressful to the parent. In many ways this mirrors findings regarding the evolution and maintenance of sexual reproduction. Both theory [59–61], and experimental evolution studies (Morran et al., 2009) demonstrate that in fluctuating and uniquely stressful environments, sexual reproduction provides fitness benefits relative to asexual reproduction. The access to a wider range of phenotypic space afforded to the offspring of sexually reproducing individuals, or individuals transmitting variable epigenetic profiles, may prove advantageous under specific conditions of environmental stress and/or fluctuation.

Initially epigenetic variability induced by stressful environments would be random, with benefits tied solely to increased phenotypic variance. It is, however possible that over time selection could favor consistent epigenetic induction at a specific locus or loci [62]. Alternatively, increased epigenetic variability in response to parental stress may be an accidental and perhaps maladaptive by-product of a loss of stasis. Evolutionary modeling, molecular epigenetic studies, and comparisons of fitness variability in the offspring of high and low stress individuals will shed light on the prevalence of stress-induced transgenerational variability, its molecular basis, and potential adaptive nature.

### CHH methylation is most responsive to parental leaf damage

*Mean* methylation was similar in offspring of damaged and control parents for over 99% of the genome (Fig. 3). Interspersed across the genome however, were parent environment-induced DMRs ranging in size from tens to thousands of base pairs. Within these regions we find consistent shifts in percent cytosine methylation in response to parental damage (Figs. 3 and 4). A clear pattern is that CHH methylation is the most responsive to parental stress (Fig. 3), with thousands of CHH DMRs scattered across the genome. However, as discussed below, the role of these CHH DMRs in gene regulation is unclear.

### Differential non-CG methylation is most strongly associated with transposable elements

Non-CG DMRs overlapped most often with TEs (Fig. 5). Speficially, CHH regions up-methylated in response to parent damage overlapped with TEs more frequently than down-methylated regions. The impact of observed TE differential methylation on gene expression remains unclear. We did not find significant enrichment of differential CHH methylation near genes previously identified as differentially expressed in response to parent damage.

Miniature inverted-repeat TEs (MITEs) in the Mutator-like elements (MULEs) superfamily were particularly likely to show a pattern of increased methylation (Figs. 5 and 6). MITEs are small TEs found at high copy-number in nearly all plants [63]. Unlike many other types of TEs often located near centromeres in highly repetitive heterochromatic regions, MITEs tend to be located in gene-rich euchromatic regions and generate a large number of 24-nt si-RNAs [64]. 24-nt si-RNAs are responsible for RNA-dependent DNA methylation, suggesting a mechanism for the enrichment of methylation at these loci. If damage induces increased 24-nt si-RNA production at MITE loci, this may lead to their differential methylation in the next generation as 24nt si-RNAs are known to be phloem mobile [38] and capable of infiltrating the germline [41]. Additionally, recent work has demonstrated that parental and grandparental environment can leave a lasting impact on small-RNA profiles [65]. Therefore, methylation of these regions could be re-programmed through the action of si-RNAs after demethylation that occurs during germline formation.

### Differential CG methylation is strongly associated with gene coding sequences

Unlike changes in non-CG methylation which most often coincided with TEs and only overlapped coding regions 22% of the time, CG DMRs most frequently overlapped with protein coding regions (60.6%, Fig. 5). This suggests that differential CG methylation is more closely tied than differential non-CG methylation to gene regulation. The types of genes that were differentially methylated were not a random subset of the transcriptome, but were enriched for genes in specific gene ontology categories. Genes overlapping with both CG and non-CG DMRs were enriched for genes related to plant hormone regulation, and in just the set of CG DMRs, we found enrichment for genes related to responses to external stimuli.

Taken into consideration alongside the enrichment of CG-DMRs in coding sequences genome wide, these results suggest that the altered methylation of genes may lead to an epigenetic memory in offspring that affects their interactions with the environment. A recent study in *Arabidopsis* demonstrated that histone variant H3.3 leads to an increase of gene body DNA methylation, a depletion in H1, and the differential expression of a subset of genes [66]. Interestingly, this group found that H3.3 knockdown lead to the differential expression of genes enriched for hormone and stimulus response associated genes [66], similar to the types of genes found differentially methylated here, and previously identified as differentially expressed [4]. This subset of the genome regulated by histone variant H3.3 may be both environmentally responsive, and prone to persistence across generations.

### Targets of transgenerational epigenetic plasticity

Table 3 highlights 12 genes that exhibited differential CG methylation, were identified in our prior work as differentially expressed in response to parental damage, and have close homologs in model species with well-defined functions. These genes span diverse functions; seven code for enzymes, and five putatively function in protein-protein interactions or nucleotide binding. Pectin lysase functions in cell-wall metabolism. Its increased expression in response to parental damage may relate to cell wall breakdown during trichome development. Cytosolic sulfotransferase 12 negatively regulates brassinosteroid 24-epicathasterone activity [67]. Brassinosteroids both positively [68] and negatively [69] regulate trichome production. Therefore, differential methylation and expression of genes involved in brassinosteroid production and pectin lysase make interesting candidates for transgenerational trichome induction.

**Table 3:** Twelve candidate genes that overlapped with a coding region CG DMR and were previously found to be differentially expressed

Gene	Function	Direction Methylation	Direction Expression
Migut.L01898	Cytochrome P450	Down (32%)	Up
Migut.L01459	Pectin Lysase	Down (43%)	Up
Migut.N01808	Pheophytinase	Down (24%)	Down
Migut.A00087	Thermospermine Synthase	Down (44%)	Down
Migut.N023227	Homeobox	Down (55%)	Down
Migut.H00566	*Constans*-like protein	Up (18%)	Up
Migut.M01851	B-Box Zinc Finger	Up (53%)	Up
Migut.J01585	MATE efflux pump	Up (61%)	Up
Migut.N01811	CCH Zinc Finger	Up (25%)	Down
Migut.L01164	Sulfotransferase	Up (38%)	Down
Migut.N0224	LRR-Kinase	Up (42%)	Down
Migut.N01831	Aspartic Protease	Up (47%)	Down

Of the remaining five enzyme-coding genes, two have possible roles in plant hormone synthesis, two in the breakdown of compounds, and one as a transport protein. Of those involved in hormone synthesis, a 71 subgroup cytochrome P450 was up-regulated, while thermospermine synthase was down-regulated. Subgroup 71 cytochromes are stress-inducible and involved in jasmonic acid synthesis [70]. Thermospermine synthase produces thermospermine, a growth regulator involved in xylem differentiation. Interestingly, thermospermine synthase acts on a precursor in the synthesis of ethylene, spermine, and thermospermine. There is evidence that numerous enzymes compete for this precursor [71]. Through reduced thermospermine synthase, there is potentially increased flux into the production of spermine and/or ethylene, both involved in plant stress responses. The differential methylation and expression of a pheophytinase and an aspartic protease suggest that catabolism of proteins and chloroplasts may be altered by parental damage. The differential methylation of a MATE efflux protein is an intriguing candidate; this class of proteins is known to be vital in the transport of plant hormones, secondary metabolites, and other organic compounds [72].

The five differentially methylated, differentially expressed regulatory genes provide intriguing candidates as upstream elicitors of transgenerational plasticity. Two B-box zinc finger genes showed increased methylation and increased expression in response to parental damage. Three additional proteins with a regulatory capacity showed decreased expression. While the function of regulatory genes is difficult to determine through orthology assessment, the leucine rich repeat kinase is closely related to *Arabidopsis* NILR1, one of the most well studied genes involved in activating defense responses to pathogens [73]. In *Arabidospsis,* this gene detects pathogen-associated molecular patterns, initiating the synthesis of host-resistance (R-genes). R-genes are known to be transgenerationally regulated, both via priming as well as constitutive induction in response to parental pathogen infection [74]. While to our knowledge, all previous examples have demonstrated epigenetic up-regulation in response to pathogen response, this finding suggests that in response to other stresses R-genes may be epigenetically down-regulated. A tradeoff between microbial and herbivore defense may shift the optimal allocation of resources away from pathogen defenses in an enviornment of high herbivory.

### Association between differential methylation and gene expression

Our current methylation and previous gene expression data come from different plants, making it impossible to directly compare methylation and gene expression levels in an individual plant. The current data is therefore insufficient to thoroughly evaluate the effect of methylation on gene expression in response to parental environment. While the separation of experiments undermines power to detect methylation-to-expression effects (false negatives inflated), it should not create false positives. We are afforded less insight into the subtleties of the impact of DNA methylation on gene expression, but we gain confidence that loci identified in both experiments are consistent targets of transgenerational epigenetic inheritance. Therefore, despite reduced power, a preliminary comparison is useful.

Nearly three times as many genes overlapping CG DMRs were previously identified as differentially expressed (16) [4] compared to the null expectation (5.6) (Fig. 5). This significant overlap of CG DMRs within gene bodies adds to a growing body of evidence [66, 75] for an interaction between gene body CG methylation and gene regulation. Not only were CG DMRs more likely than non-CG DMRs to overlap with coding sequences, but CG DMR genes were more likely to be differentially expressed than non-CG DMR genes, or genes not overlapping with any DMRs. We consider our results suggestive, yet inconclusive evidence that differential methylation is linked to patterns of transgenerational plasticity.

While we found a tentative association between differential gene body CG methylation and differential gene expression in the offspring of damaged plants, we did not find a direct correlation between the direction of change in gene expression and gene body CG methylation. A likely explanation stems from the complex relationship between coding sequence methylation and gene expression. In our prior modeling of the relationship between DNA methylation and gene expression in *M. guttatus*, we identified that along with significant first, second, and third order CG coding sequence methylation effects on gene expression, CG coding sequence methylation interacted with 6 other model terms to alter gene expression (Colicchio et al., [4]). Zilberman [35] and Bewick and Schmitz [31] argue that there is no clear direct role of gene body methylation on gene regulation from either a molecular or evolutionary perspective. They suggest alternative roles including differential gene body CG methylation enhancing splicing accuracy [76], inhibiting RNA polymerase II activity [28], displacing variant histone H2A.Z [77], or potentially resulting from local adaptation through some as of yet unknown mechanism [78]. Additionally, complex feedback loops between H3.3, H1, H2A.Z, small RNA production, and DNA methylation likely play a role in modifying or sustaining gene expression across environmentally responsive genes which obfuscates a direct link between levels of CG methylation and gene expression [66]. It will require studies with the same plants analyzed for gene expression, methylation, and other epigenetic markers in response to environmental perturbations to gain insights into the link between gene body CG methylation and gene expression.

## Conclusions

The transmission of epigenetic marks between generations represents one mechanism for maintaining environmentally induced gene expression after an initial signal recedes. We assayed DNA methylation variation dependent on parental environment and found differential CG and non-CG methylation across gene coding regions and TEs, respectively. The enrichment of genes previously identified as differentially expressed in response to parental damage that overlaped CG DMRs provides suggestive evidence that inheritance of altered methylation profiles is associated with transgenerational gene expression plasticity. Recent work has demonstrated a complex relationships between histone modifications and variants and DNA methylation, and their combined effects on gene expression [66]. Thus, the differential DNA methylation patterns documented here represent a snapshot of how parent damage leads to alteration in one component of the epigenome. As this study was preformed in the direct offspring of wounded plants it is possible that observed differences in DNA methylation are a product of damage induced maternal effects such as seed priming [79], rather than being directly inherited as altered epigenetic profiles from the parental germ line. Evidence in *Mimulus* demonstrates that transgenerational phenotypic effects persist for multiple generations [45], suggesting that altered epigenetic profiles may contribute to offspring phenotype. However, future studies are necessary to confirm this multi-generational epigenetic persistence and to determine whether herbivory directly alters the methylome of parental *Mimulus* individuals.

The precise role of differential gene body methylation for transgenerational plasticity, and genome regulation more generally, remains a critical questions in epigenetics [31, 35]. Beyond a relatively small subset of the genome exibiting targeted differential methylation, we identified genome-wide increases in methylome variation and differential methylation of specific TE classes. This suggests that parental conditions can alter an organisms epigenetic profile in a host of ways. Of particular evolutionary significance, increased epigenetic diversity in progeny may be an unintended side effect of a parental environment, and may increase offspring phenotypic variance in a manner potentially adaptive under stressful conditions. Together, our results move us closer to deciphering the mechanism(s) through which parental environment may adaptively tune offspring development.

## Additional files

Additional file 1: **Table S1.** Re-annotated gene positions for RIL 94 genome. (XLSX 1025 kb)

Additional file 2: **Table S2.** Re-annotated transposable element positions for RIL 94 genome. (XLSX 12403 kb)

Additional file 3: **Figure S1.** Methylation domain landscape (MDL) plots showing the distribution of methyl-regions across the genome. X-axis shows the log-transformed genomic region size, while the Y-axis shows the percent methylation within the given region, density represents the number of methylated regions of a given size. **Figure S2.** Patterns of average CG, CHG, and CHH methylation in and around genes. Genomic regions up-stream (5’) of coding regions shown as negative distances, bases within coding regions are in the grey box and displayed as a percentage between the 5’ and 3’ end of the gene, and regions down-stream (3’) coding regions shown as positive distances. **Figure S3.** Desnity plot showing the distribution of absolute percent change differences in our 3 classes of DMRs. **Figure S4.** General patterns of overlap between genes and CG/non-CG methylation. **Figure S5.** Overview of the top 15 CG DMRs by statistical significance overlapping genes. **Figure S6.** Venn diagram showing overlap between coding regions of genes from the older *M*. *guttatus* annotation (bounded in red) used in Colicchio et al., 2015b. Differentially expressed genes identified from this older annotation (DE), and differentially methylated regions (CG DMR, CHG DMR and CHH DMR) identified in this study using a newer *M. guttatus* annotation (https://phytozome.jgi.doe.gov/pz/portal.html#!info?alias=Org_Mguttatus) are shown (bounded in blue). **Figure S7.** Visualization of two differentially CG methylated coding regions that overlap with genes identified as down-regulated in Colicchio *et al.* (2015b). **Methods S1.** Methods involved in the construction of the new RIL 94 reference genome. (DOCX 1824 kb)

Additional file 4: **Table S3.** Positions of differentially methylated regions, class and direction of change, and their proximity to transposable elements and genes. (XLSX 459 kb)

Additional file 5: **Table S4.** GO term enrichment of genes that overlap with CG (a) and non-CG DMRs. (XLSX 62 kb)
