# Supplementary material for: Parental experience modifies the Mimulus methylome
Source: BMC Genomics. 2018 Oct 12;19:746. doi: 10.1186/s12864-018-5087-x (PMC6186029; doi:10.1186/s12864-018-5087-x)
Supplement: Supplementary file 3 — Figure S1. Methylation domain landscape (MDL) plots showing the distribution of methyl-regions across the genome. X-axis shows the log-transformed genomic region size, while the Y-axis shows the percent methylation within the given region, density represents the number of methylated regions of a given size. Figure S2. Patterns of average CG, CHG, and CHH methylation in and around genes. Genomic regions up-stream (5’) of coding regions shown as negative distances, bases within coding regions are in the grey box and displayed as a percentage between the 5’ and 3’ end of the gene, and regions down-stream (3’) coding regions shown as positive distances. Figure S3. Desnity plot showing the distribution of absolute percent change differences in our 3 classes of DMRs. Figure S4. General patterns of overlap between genes and CG/non-CG methylation. Figure S5. Overview of the top 15 CG DMRs by statistical significance overlapping genes. Figure S6. Venn diagram showing overlap between coding regions of genes from the older M. guttatus annotation (bounded in red) used in Colicchio et al., 2015b. Differentially expressed genes identified from this older annotation (DE), and differentially methylated regions (CG DMR, CHG DMR and CHH DMR) identified in this study using a newer M. guttatus annotation (https://phytozome.jgi.doe.gov/pz/portal.html#!info?alias=Org_Mguttatus) are shown (bounded in blue). Figure S7. Visualization of two differentially CG methylated coding regions that overlap with genes identified as down-regulated in Colicchio et al. (2015b). Methods S1. Methods involved in the construction of the new RIL 94 reference genome. (DOCX 1824 kb) [file 12864_2018_5087_MOESM3_ESM.docx]

**Figure S1.** Methylation domain landscape (MDL) plots showing the distribution of CG methyl-regions across the genome. X-axis shows the log-transformed genomic region size, while the Y-axis shows the percent methylation within the given region, density represents the number of methylated regions of a given size.

**Figure S2.** Patterns of average CG, CHG, and CHH methylation in and around genes. Genomic regions up-stream (5’) of coding regions shown as negative distances, bases within coding regions are in the grey box and displayed as a percentage between the 5’ and 3’ end of the gene, and regions down-stream (3’) coding regions shown as positive distances.

**
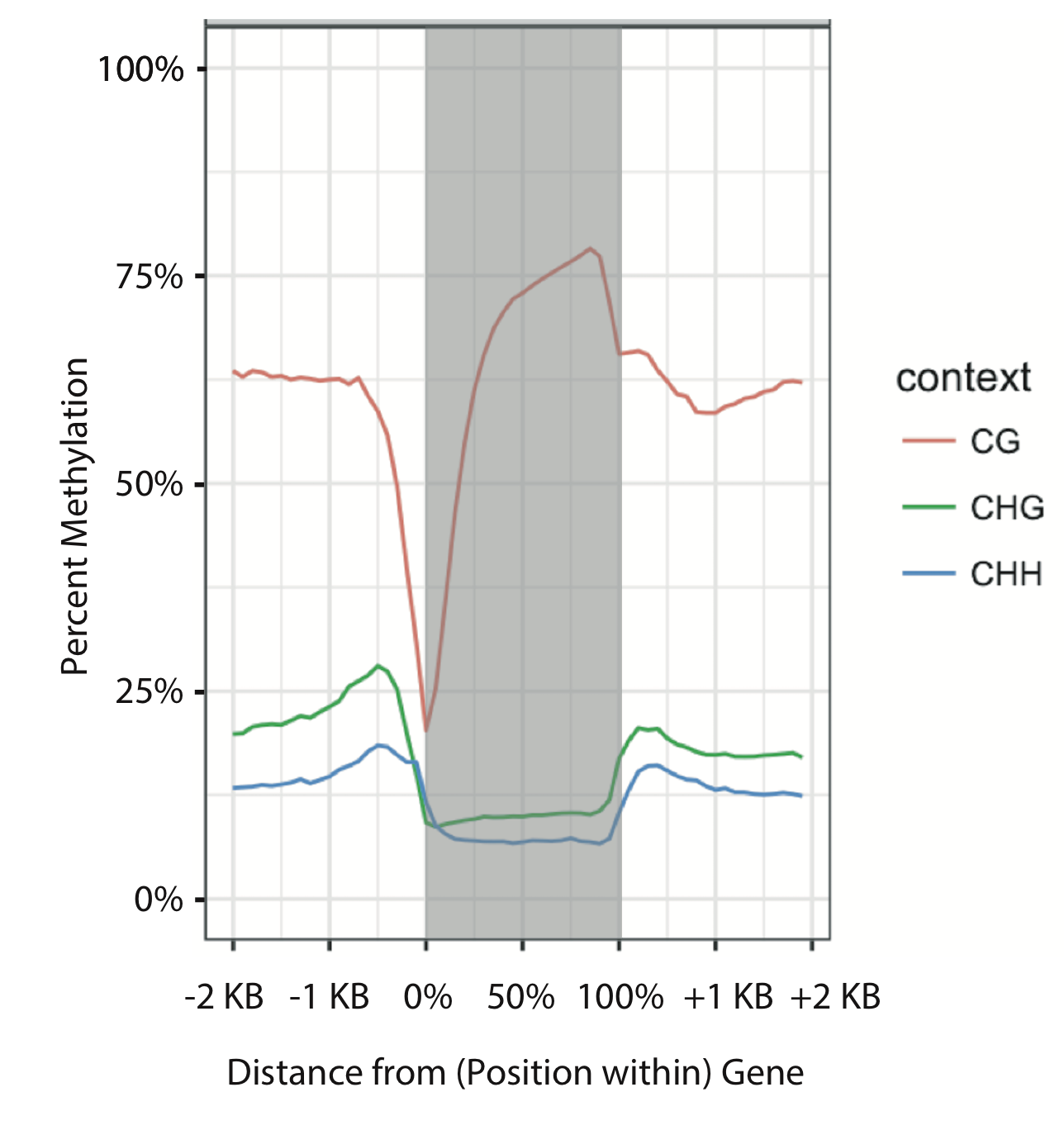
**

**Figure S3.** Desnity plot showing the distribution of absolute percent change differences in our 3 classes of DMRs.


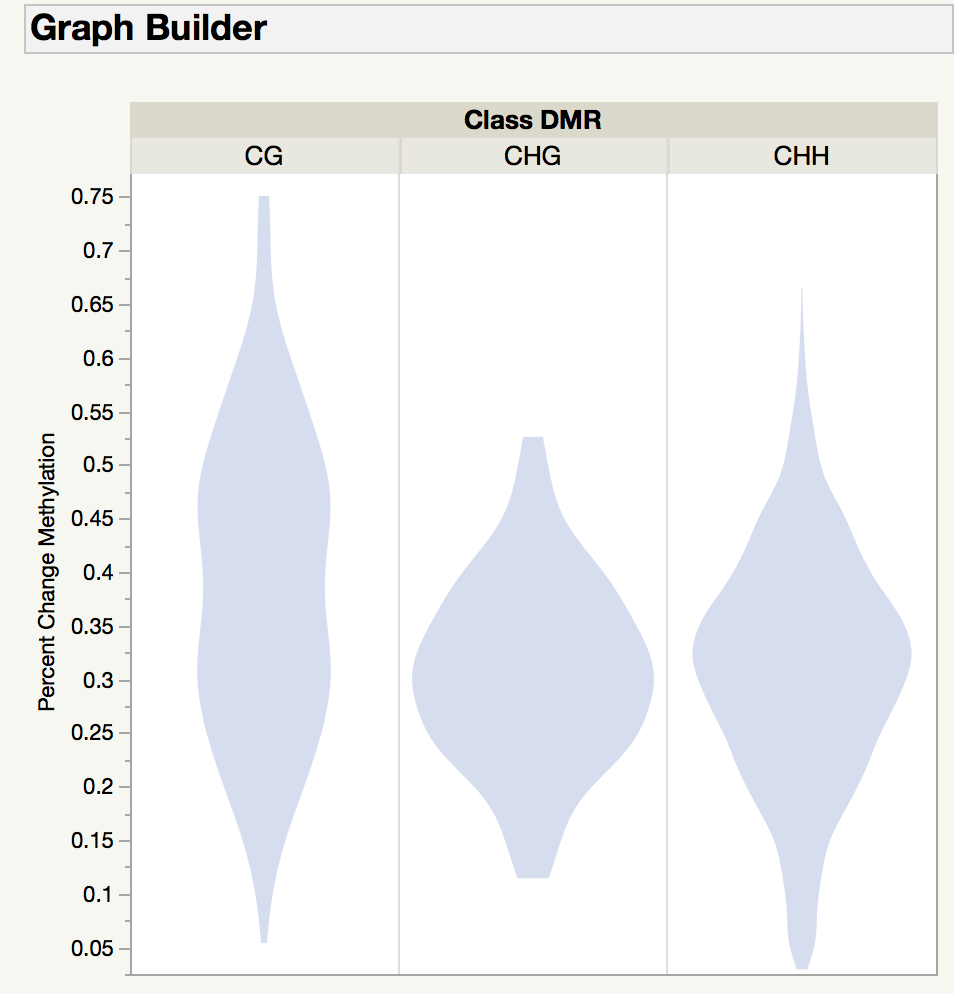


**Figure S4.** General patterns of overlap between genes and CG/non-CG methylation. (A) CG methylation was enriched in genes; non-CG methylation was enriched in transposable elements. (B) CG methylation was enriched in gene body coding sequences previously identified as differentially expressed in Colicchio *et al.* (2015b). Grey shading represents expected number of differentially expressed genes based on random chance.

**
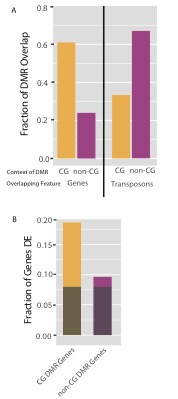
**

**Figure S5.** Overview of the top 15 CG DMRs overlapping genes. In right-side panels for each gene, the region bounded by black bars represent the location of the gene, while the region bounded by blue bars demarcates the DMR. In the left-side panel for each gene, % methylation within the DMR is shown for the progeny of damaged plants (red) and progeny of control plants (green).

**
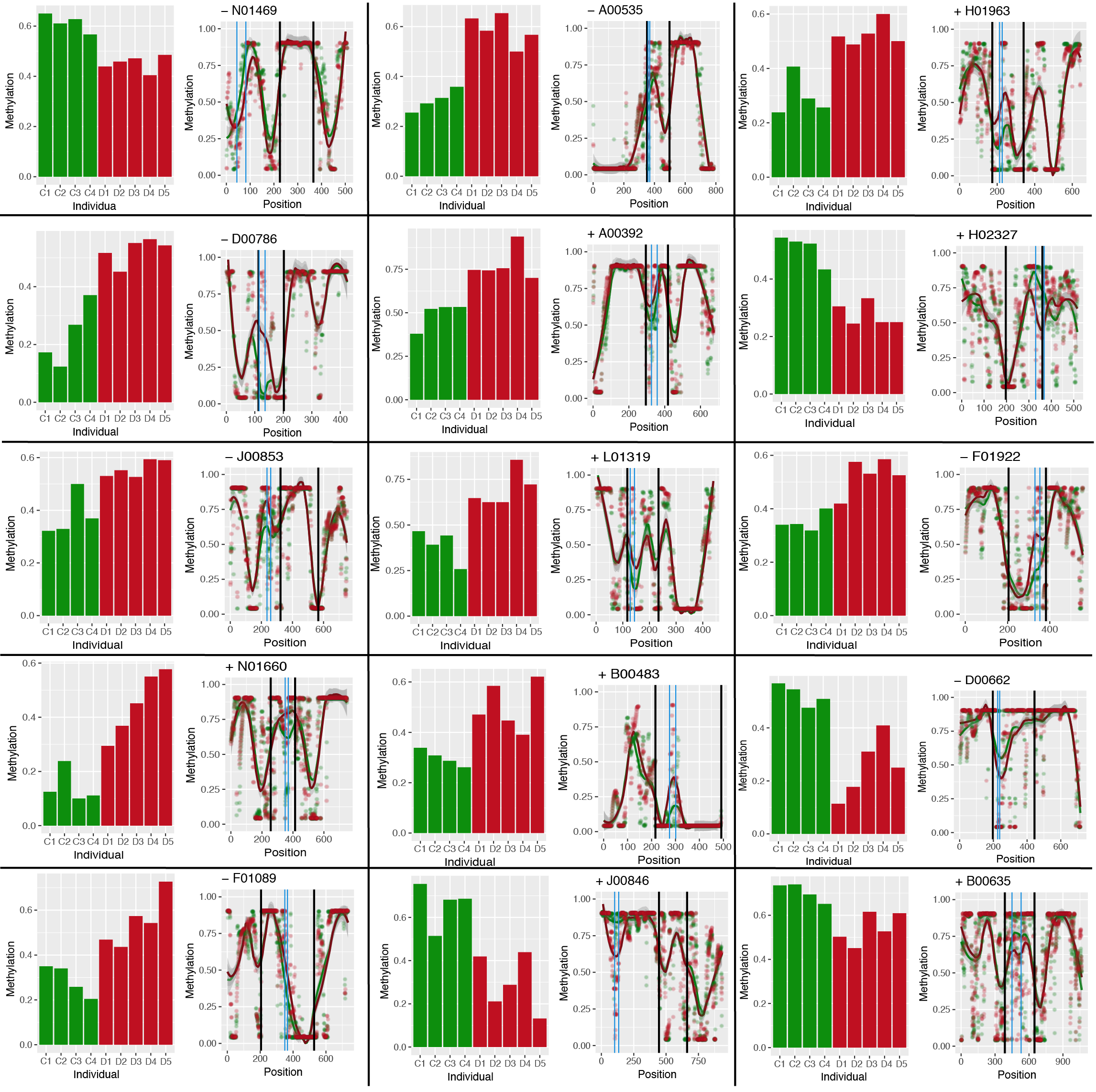
**

**Figure S6.** Venn diagram showing overlap between coding regions of genes from the older *M*. *guttatus* annotation (bounded in red) used in Colicchio et al., 2015b. Differentially expressed genes identified from this older annotation (DE), and differentially methylated regions (CG DMR, CHG DMR and CHH DMR) identified in this study using a newer *M. guttatus* annotation (https://phytozome.jgi.doe.gov/pz/portal.html#!info?alias=Org_Mguttatus) are shown (bounded in blue).


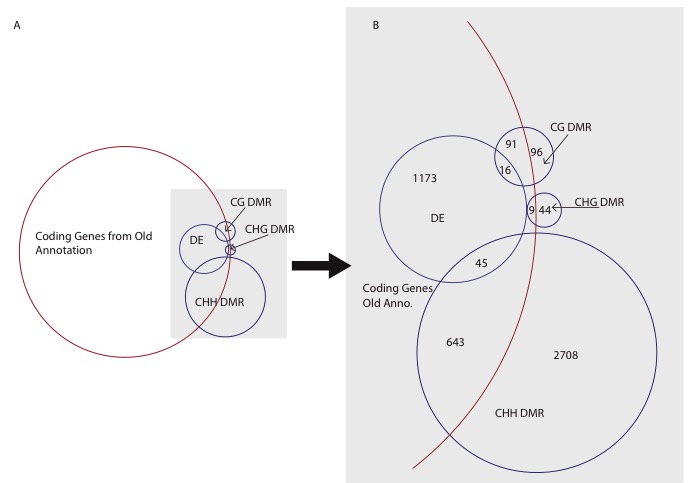


**Figure S7.** Visualization of two differentially CG methylated coding regions that overlap with genes identified as down-regulated in Colicchio *et al.* (2015b). (A) Thermospermine Synthase. A DMR near the 5’ end of the gene is down-methylated with parent damage. The bottom panel (left) provides raw methylation data within the DMR, which was the input to the GLM to determine significance. The bottom panel (right) shows average gene expression for Thermospermine Synthase in offspring of damage and control parents. (B) Aspartic Protease methylation data presented as in (A). A DMR near the 5’ end of the gene is up-methylated with parent damage.


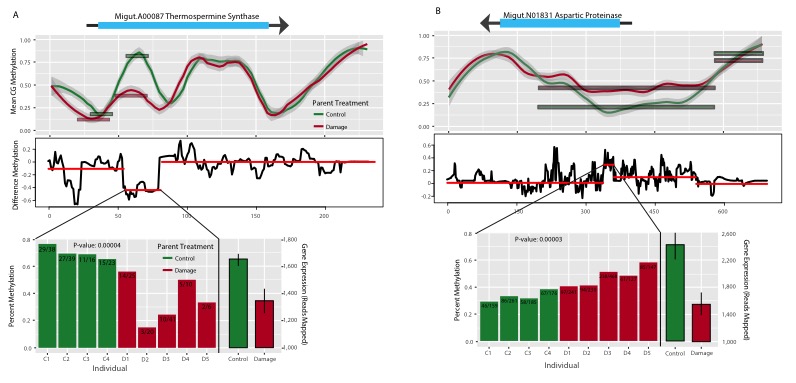


**Methods S1.** **Reference Genome Construction:** We extracted genomic DNA from a single RIL94 plant using a CTAB protocol (Holeski *et al.*, 2013). We prepared a genomic DNA library with the Illumina Nextera kit (Illumina Inc., San Diego, CA) and sequenced on single HiSeq 2500 101 bp, paired end lane obtaining 39,257,138 read pairs (ca. 17x coverage of the genome). We mapped read pairs to the *M. guttatus* v2.0 draft genome with organelle genomes included (<http://www.phytozome.net/>) using the BWA (<http://bio-bwa.sourceforge.net/>) mem command followed by SAMtools (<http://samtools.sourceforge.net/>). We used Picard tools (<http://broadinstitute.github.io/picard/>) to add ReadGroups to the resulting bam files and then indexed these files using SAMtools. Next, we used CLC's extract consensus sequence tool, inserting Ns for ambiguous sequences, and other default settings to create a RIL 94 fasta reference file. We used an existing *M. guttatus* annotation to locate the position of annotated genes in our RIL 94 reference genome. We manually converted the *M. guttatus* reference IM62 gff and IM62 fasta files (from Phytozome: https://phytozome.jgi.doe.gov/pz/portal.html) to a bed file in R. Next, bedtools getfasta was used to capture the nucleotide sequences of individual genes from the IM62 sequence into a fasta file. BWA mem was then used to map each gene in the IM62 genome to the RIL 94 reference genome. This generated a bam file which we converted into a bed file using bedtools bamtobed. We manually converted this bed file into a gff in R (Ihaka & Gentleman, 1996). Approximately 99% of IM62-derived genes mapped to the same scaffold, in the same orientation, and within 10% of their starting position when mapped to the RIL 94 reference. These mapped gene coordinates were used for subsequent analyses (Supplemental Table 1). To locate the position of transposable elements (TEs) in the RIL 94 reference genome, we used the curated TE library (Edger *et al.*, 2017) followed by repeatmasker. We then used the table output to annotate the class, family, and position of TEs across the genome (Supplemental Table 2).

**Read Mapping:** We constructed a RIL 94 reference genome for methylome read mapping analyses We then used the software BMap (Miura et al., 2012) (http://itolab.med.kyushu-u.ac.jp/BMap/index.html) to map bisulfite treated reads to the RIL 94 reference genome. In short, BMap first searches candidate genomic loci for each read in two duplicated genome sequences using an approach called adaptive seed (Kiełbasa et al., 2011). Next, BMap creates pairwise alignments between the read and original DNA sequence at each candidate locus, and reports alignments with the highest score for each read. We used default parameters for mapping with BMap, as previously optimized (Colicchio *et al.*, 2015). Using alignments exported by BMap, methylation status for every cytosine in every read was called and counts both supporting the methylated and unmethylated state were assigned for every cytosine residue of the reference genome. After calculating the raw number of methylated and unmethylated cytosines using the Bmap approach, we used the R package methimpute, to more accurately assess individual base pair percent methylation across our nine individuals. We loaded raw methylome data into R as a data frame with a column for chromosome, position, number of reads mapped, number of reads methylated, and methylation context (CG, CHG, CHH). We converted this file to a Genomic Ranges file using the GRanges function. Next we used methimpute with mode separate.contexts=T to calculate average methylation across our 5 parental wounding individuals and our 4 control individuals to establish mean methylation levels at each cytosine for each treatment.

**References:**

**Colicchio JM, Miura F, Kelly JK, Ito T, Hileman LC**. **2015**. DNA methylation and gene expression in Mimulus guttatus. *BMC Genomics* **16**: 507.

**Edger PP, Smith RD, McKain MR, Cooley AM, Vallejo-Marin M, Yuan Y-W, Bewick AJ, Ji L, Platts AE, Bowman MJ, *et al.*** **2017**. Subgenome dominance in an interspecific hybrid, synthetic allopolyploid, and a 140-year- old naturally established neo-allopolyploid monkeyflower. *The Plant Cell*: tpc.00010.2017.

**Holeski LM, Keefover-Ring K, Bowers MD, Harnenz ZT, Lindroth RL**. **2013**. Patterns of Phytochemical Variation in Mimulus guttatus (Yellow Monkeyflower). *Journal of Chemical Ecology* **39**: 525–536.

**Ihaka R, Gentleman R**. **1996**. R: A Language for Data Analysis and Graphics. *Journal of Computational and Graphical Statistics* **5**: 299–314.
